# Study on Carbonation Resistance of Polymer-Modified Sulphoaluminate Cement-Based Materials

**DOI:** 10.3390/ma15238635

**Published:** 2022-12-03

**Authors:** Ping Zhang, Bingxin Zhang, Yanfeng Fang, Jun Chang

**Affiliations:** 1 School of Materials and Architectural Engineering, Guizhou Normal University, Guiyang 550025, China; 2 School of Materials Science and Engineering, Shenyang Jianzhu University, Shenyang 110168, China; 3 School of Civil Engineering, Dalian University of Technology, Dalian 116024, China

**Keywords:** sulphoaluminate cement, carbonation, polymer, polycarboxylic acid, carbonation level

## Abstract

The use of tricyclic copolymer latex (AMPS) can effectively improve the carbonation resistance of sulphoaluminate cement. This paper investigated polymer AMPS and polycarboxylic acid to modify sulphoaluminate cement materials by exploring the carbonation level of sulphoaluminate cement paste and mortar and the strength before and after carbonation. Then, the optimal dosage of polymer and polycarboxylic acid was obtained so that the carbonation resistance of sulphoaluminate cement reached the best state. The compressive strength was significantly improved by adding AMPS for sulphoaluminate cement paste and mortar. After carbonation, the strength decreased and combined with the carbonation level; it was concluded that the carbonation resistance of sulphoaluminate cement materials was the best when the optimal dosage of AMPS and polycarboxylic acid was 5% and 1.8%, respectively. Due to the addition of AMPS, the hydrated calcium aluminosilicate (C-A-S-H) and hydrated calcium silicate (C-S-H) gels, generated by the hydration of sulphoaluminate cement and the surface of unreacted cement particles, are wrapped by AMPS particles. The water is discharged through cement hydration. The polymer particles on the surface of the hydration product merge into a continuous film, which binds the cement hydration product together to form an overall network structure, penetrating the entire cement hydration phase and forming a polymer cement mortar with excellent structural sealing performance. To prevent the entry of CO_2_ and achieve the effect of anti-carbonation, adding polycarboxylic acid mainly improves the sample’s internal density to achieve the anti-carbonation purpose.

## 1. Introduction

With the acceleration of China’s modernization process, various infrastructures have been constructed. Most of these are reinforced concrete structures. In the design process, the durability, practicability, and comfort of these structures should also be considered in addition to their traditional mechanical properties, such as strength, earthquake resistance and low-carbon [1,2]. Concrete is the most widely used artificial material and an essential building material. With the increase in the service life of concrete, the design life of the structure will often be lower than its service life and it may be destroyed prematurely. This arouses people’s attention and research on structural durability, something which has become a common concern in the engineering field. The durability of concrete refers to a property in which the concrete maintains the original mechanical properties and changes slightly during the service process of the structure [1,3,4]. The durability of concrete is closely related to its internal and external environment. Internal factors include concrete strength, permeability, the thickness of the protective layer, cement type, cement label, cement dosage, admixture dosage, etc.; external factors mainly include ambient temperature, humidity, carbon dioxide content, and corrosive environment. The durability problems of concrete in practical applications mainly include [5,6]: (1) carbonation of concrete and corrosion of steel bars; (2) freeze–thaw damage of concrete; (3) erosion of chemical medium; (4) corrosion of physical concrete wear; (5) alkali–aggregate reaction [7,8]. Among the many durability problems, more attention has been paid to concrete carbonation [9,10,11]. Acid gases in the air, such as CO_2_, HCl, SO_2_, Cl_2_, and others, dissolve in water and enter the component’s interior. Reacting with the alkaline substances after the cement hardens is called the neutralization of concrete [12,13,14].

In some cases, the carbonation of concrete can increase the internal compactness, thereby improving the concrete’s resistance to chemical attack [15,16,17]. However, carbonation will reduce the alkalinity of concrete, destroy the passivation film on the surface of the steel bar, and make the concrete lose its protective effect on the steel bar, which harms the corrosion of the steel bar in the concrete. At the same time, concrete carbonation will also increase the shrinkage of concrete, which may lead to substantial cracks and structural damage [18]. Since concrete is a loose and porous material, there are capillaries, pores, air bubbles, and even defects of different sizes. The CO_2_ gas in the air penetrates the pores of the concrete and chemically reacts with the carbonate substances in the pores. Carbonate substances are produced in the hydration process of sulphoaluminate cement, mainly AFt, AFm, and gels C-A-S-H, C-S-H, and aluminum hydroxide gel (AH_3_) produced by hydration [19,20,21].

**Figure 1:**
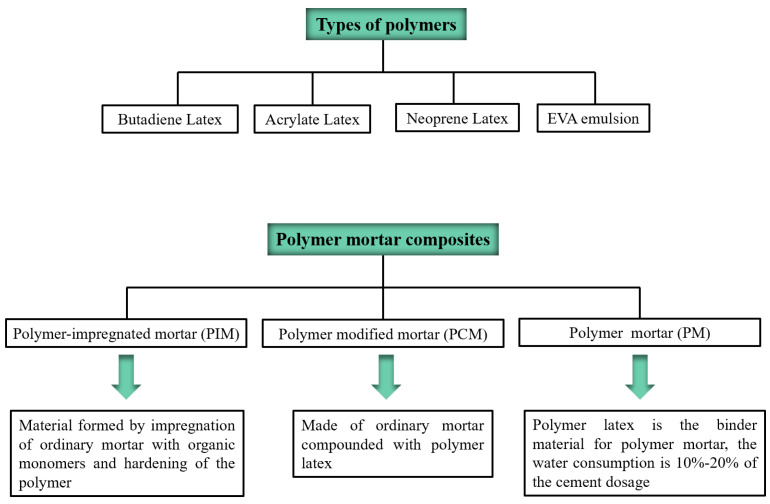
Types of polymers and classification of polymer mortar composite materials.

**Figure 2:**
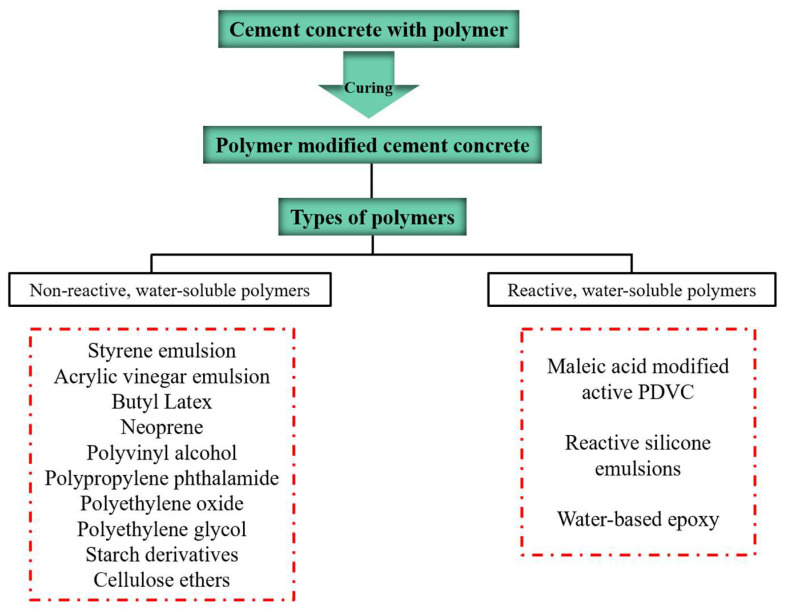
Classification of polymers used in polymer-modified cement concrete.

In recent years, using organic polymers as part of cementitious materials in the preparation of polymer-modified concrete mortar has been a critical way to improve the tensile strength, toughness, and impermeability of cement-based materials [22]. Tests show that a variety of replacement polymers forms part of the cement. After being mixed into the cement mortar, the compressive and flexural strength of the polymer cement mortar is significantly enhanced, the toughness of the cement-based material is improved considerably, and the air tightness is increased, thereby achieving carbonation resistance [23]. Figure 1 and Figure 2 show the polymers and polymer mortars used in existing cement concrete-modified materials.

When the polymer emulsion is mixed with the cement mortar, the emulsion is uniformly dispersed in the cement mortar. With water loss from the cement paste, the hydration products AFt, AFm, and the gels C-A-S-H, C-S-H, and aluminum hydroxide gel (AH_3_) produced by hydration gradually aggregated. At the same time, the polymer particles were partially deposited in the gel and untreated and hydrated cement particle mixture surface. The polymer particles on the surface of the cement hydration product merge into a continuous film, which binds the cement hydration product together to form an overall network structure. The film formed by the polymer in the network runs through the entire cement hydration product, creating a dense structural polymer cement mortar [24]. Sulphoaluminate cement, as a kind of low-carbon cement, has a wide range of market application prospects [25,26,27].

In this experiment, AMPS polymer emulsion and polycarboxylic acid were selected as modified substances to be mixed into sulphoaluminate cement paste, mortar, and concrete to enhance the compactness of cement and achieve the purpose of anti-carbonation. This paper’s research focuses on the carbonation resistance of sulphoaluminate cement, including: the carbonation mechanism of polymer-modified sulphoaluminate cement; change in compressive strength before and after carbonation; determination of carbonation depth and carbonation area.

## 2. Raw Materials and Experimental Methods

### 2.1. Raw Materials

Sulphoaluminate cement is mainly composed of 3CaO·3Al_2_O_3_·CaSO_4_ minerals. The cement has excellent properties, such as early strength, high strength, frost resistance, impermeability, corrosion resistance, and low alkalinity [28,29,30]. The 42.5 fast-hardening sulphoaluminate cement used in this study was produced by Tangshan Polar Bear Building Materials Co., Ltd. (Tangshan, China). In order to compare the carbonation resistance of sulphoaluminate cement (SAC), 42.5 ordinary Portland cement was also used in the test. Table 1 shows the elements and properties of sulphoaluminate cement and ordinary Portland cement (OPC).

In this experiment, 3% terpolymer latex (AMPS) was used and its solid content was 38.16%, which is produced by Changshu BATF Technology Co., Ltd. (Changshu, China). The polymer’s ionic stability, mechanical stability, centrifugation stability, dilution stability, and storage stability all meet the requirements. The polymer does not stratify after being placed for a long time and the viscosity does not change significantly. SR3 is a polycarboxylate superplasticizer produced by Chongqing Sansheng Industrial Co., Ltd. (Chongqing, China), with a water-reducing rate of 30%, which is used in the experiment to design the molecular structure of the carboxylic acid copolymer according to the principle of cement dispersion. The molecular structure of this type of water-reducing agent is comb shaped. Therefore, the water-reducing agent does not fall off from the particle surface with the progress of cement hydration, which helps the cement paste to have good fluidity for a long time [31].

### 2.2. Experimental Methods

**Figure 3:**
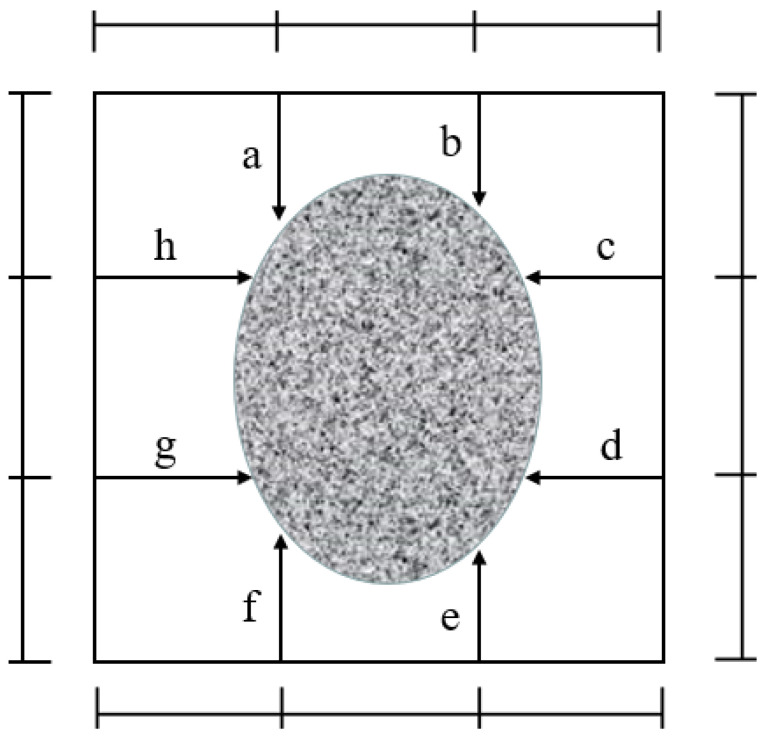
Measurement method of carbonation depth.

Through this experimental research on the carbonation depth and strength of the sulphoaluminate cement paste and mortar mixed with a polymer, and compared with ordinary Portland cement, the anti-carbonation performance of sulphoaluminate-cement-based materials was explored. The content of AMPS was 5%, 10%, and 20% of the mass of sulphoaluminate cement, and the content of polycarboxylate was 0.6%, 1.2%, and 1.8% of the mass of sulphoaluminate cement, respectively. The specific experimental mixture is shown in Table 2 and Table 3. The preparation method of the test piece is as follows: after weighing the specified amount, pour it into the cement paste mixer, respectively, stir the material according to the operating specifications, and then quickly put the material into the mold; while loading the fabric, use a scraper to insert the material evenly and then jump. Vibrate on the table, smooth it with a scraper, and put it into a standard curing box for curing. The formed cement paste is demolded after 1 day and then put into water for curing. The SAC specimens were cured in water for 2 days, the OPC specimens were taken out after curing in water for 27 days, and the compressive strength was measured. The prepared test block was placed in a carbonation test box for carbonation, the carbonation pressure was 5 bar (that is, 5 atmospheres), and the carbonation time was 1.5 h. The concrete carbonation test box used in the experiment is NELD-CA070. The specific practical steps in the carbonation of the test samples are as follows: (1) put the test piece into an oven to dry at 50 °C for 24 h; (2) the dried test sample is placed in the test box for carbonation; (3) after carbonation, take out the test samples. The test samples were split into two equal parts and a 1% concentration of phenolphthalein indicator was sprayed on the fracture surface. As shown in Figure 3, from purple to red, or the junction with a noticeable color change, measure the vertical distance of 8 places, calculate the arithmetic mean as the carbonation depth, and then convert it into the area.

The experimental instruments used in the experiment include: electronic scale accurate to 0.1 g; cement paste mixer; 2 cm × 2 cm × 2 cm cement paste forming mold; jumping table; standard curing oven; oven; self-made carbonized sample; compressive strength test instrument; vernier caliper; beaker; measuring cylinder; scraper; etc.

## 3. Results and Discussion

### 3.1. Cement Paste

#### 3.1.1. Compressive Strength of Cement Paste before and after Carbonation

**Figure 4:**
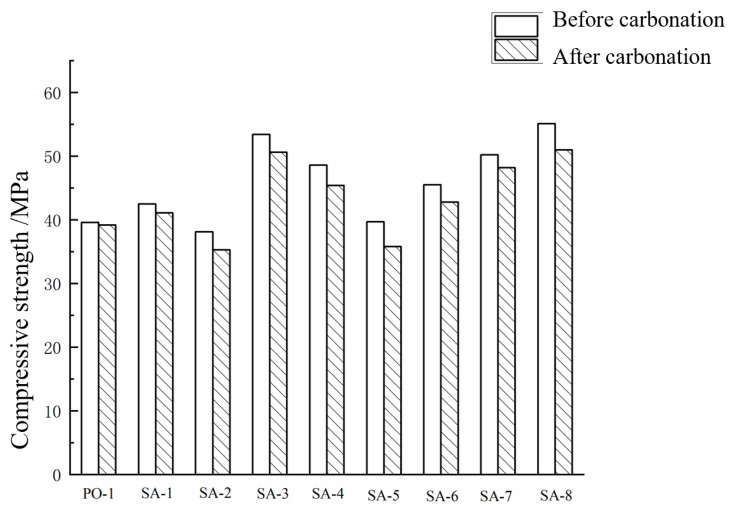
Compressive strength of cement paste before and after carbonation.

Figure 4 shows the compressive strength of the corresponding specimens before and after carbonation. Compared with the samples of PO-1 and SA-1, the sulphoaluminate cement paste with a water–cement ratio of 0.3 is compared with the Portland cement paste, and the 3-day compressive strength of the sulphoaluminate cement paste was improved. The 28-day compressive strength of sulphoaluminate cement paste is slightly higher than that of Portland cement paste, which verifies the characteristics of early strength and high strength of sulphoaluminate cement. Compared with SA-2, the compressive strength of cement paste decreased slightly with the increase in the water–cement ratio, and the strength loss rate was 10.14%. With the rise in AMPS latex content, the 3-day compressive strength of the sulphoaluminate cement paste with a water–cement ratio of 0.3 gradually decreased. Still, it was higher than the sulphoaluminate cement blank samples with a water–cement ratio of 0.35. Compared with the SA-1 blank sample, when the AMPS polymer emulsion content was 5% and 10% of the cement content, the compressive strength of the paste test block increased significantly, indicating that after the polymer emulsion was added to the cement paste, the emulsion was lost. The water forms a continuous film, and the film binds the cement hydrates together to create an overall network structure. At the same time, the polymer emulsion fills the micro-cracks inside the cement mortar, improving the specimen’s overall compactness. However, when the content of polymer emulsion is 20%, the sample’s compressive strength is lower than that of the blank group SA-1. Excessive polymer emulsion is added to the cement paste because the emulsion will lose water and form a film. The hydrated cement particles were wrapped, which hindered the cement’s hydration, so the specimen’s compressive strength was lower than that of the blank group. For sulphoaluminate cement paste, the optimum dosage of AMPS polymer emulsion is 5%. Compared with the SA-6, SA-7, and SA-8 specimens, it can be found that with the increase in the polycarboxylic acid content, the 3-day compressive strength of the sulphoaluminate cement paste with a water–cement ratio of 0.3 increases gradually and all are higher than SA-1 because adding an appropriate amount of water-reducing agent is beneficial to improve the compactness and working performance of the specimen. SA-6–SA-8 shows a trend of increasing compressive strength. The optimum dosage of polycarboxylate superplasticizer is 1.8% of the cement dosage. According to the requirements of the specification, the obtained test pieces were carbonized, respectively. It can be found that the compressive strength of each test piece after carbonation is lower than that before carbonation. Still, the changing strength trend between groups after carbonation is consistent with that before. Table 4 shows the loss rate of compressive strength of each cement paste specimen after carbonation. Among them, the compressive strength loss rate of ordinary Portland cement paste after carbonation is the smallest, which is 1.01%. The carbonation resistance of the block is better than that of sulphoaluminate cement. For the sulphoaluminate cement paste, the compressive strength loss rate of the paste with a water–cement ratio of 0.3 after carbonation is smaller than that of the paste with a water–cement ratio of 0.35. With the increase in the polymer emulsion content, the loss rate of the compressive strength of the pure paste specimens after carbonation increases gradually.

#### 3.1.2. Carbonation Depth/Area of Sulphoaluminate Cement Paste

**Figure 5:**
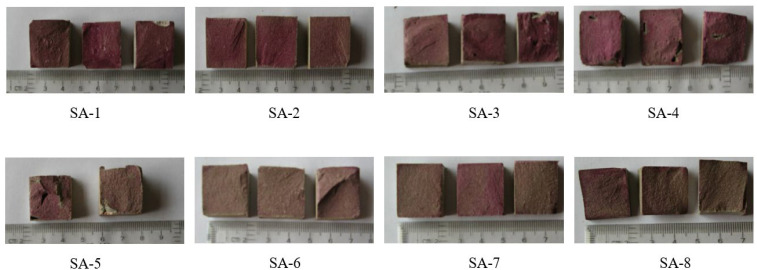
SA-1–SA-8 after carbonation with 1% concentration of phenolphthalein indicator.

The carbonized sample of the above sulphoaluminate cement paste was split into two equal parts. The phenolphthalein indicator with a concentration of 1% was sprayed on the fracture surface, as shown in Figure 5. According to the schematic diagram in Figure 3, from the surface of the specimen to the discoloration boundary, measure eight distances, obtain the arithmetic mean value as the carbonation depth h, and then calculate the carbonation area S according to Formula (1). The calculation results of the specific carbonation depth and carbonation area are shown in Table 5.

(1) $${S=20}^{2}-\;(20\;-{\;2h)\;}^{2}$$

The following conclusions can be drawn from Table 5: from the carbonation depth (h)/area (S) of the SA-3–SA-5 specimens, it can be seen that with the increase in the amount of incorporated polymer emulsion, the values of h and S gradually increased. It shows that when the amount of polymer emulsion is too great, the internal compactness of the test piece decreases, and the carbonation depth and area of the test piece increase. This phenomenon corresponds to the compressive strength of the test piece and the strength loss rate after carbonation. The carbonation depth and area of SA-3 and SA-4 are smaller than those of SA-1 and SA-2; adding an appropriate amount of polymer emulsion (5–10%) can improve the carbonation resistance of sulphoaluminate cement. Comparing the carbonation depth and area of SA-6–SA-8 specimens, it can be found that the carbonation depth/area are the largest when the polycarboxylate content is 0.6% and the minimum when the content is 1.8%. However, the carbonation depth/area of the samples under these three dosages are smaller than those of SA-1 and SA-2; adding polycarboxylic acid can improve the carbonation resistance of sulphoaluminate cement, and when the dosage is at 1.8%, the carbonation resistance is the best. 

### 3.2. Carbonation of Cement Mortar

#### 3.2.1. Compressive Strength of Cement Mortar before and after Carbonation

**Figure 6:**
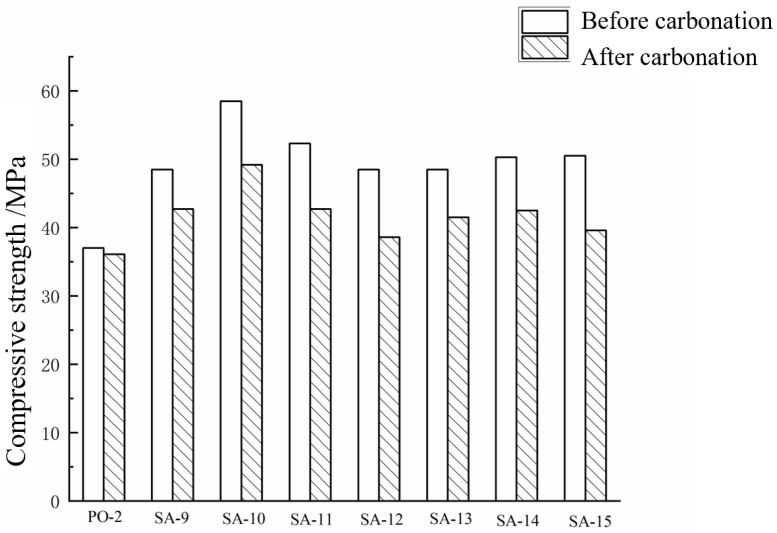
Compressive strength of cement mortar before and after carbonation.

It can be seen from Figure 6 that the 3-day compressive strength of sulphoaluminate cement mortar is 31.08% higher than that of PO-2 28-day strength; 5% AMPS polymer emulsion helps to improve the compressive strength of sulphoaluminate cement mortar, and the improvement rate is 20.62%, which is the optimal dosage of polymer emulsion under this system. After carbonating the cement mortar specimens, each specimen’s compressive strength loss rate was obtained, as shown in Table 6. After carbonation, the compressive strength of each sample decreased and the changing trend of compressive strength among the groups was consistent with the compressive strength before carbonation. In Table 6 SA-9–SA-12, the compressive strength loss rate increases gradually with the increase in polymer emulsion content. When AMPS was added with 5%, although the carbonation of the specimens had a more significant loss in strength, the strength after carbonation was higher than that of the blank sample (SA-9) before carbonation, indicating that the addition of polymer emulsions can improve the strength of sulphoaluminate and the carbonation resistance of cement mortar. The effect of polycarboxylate on the strength loss rate of sulphoaluminate cement mortar (SA-13–SA-15) after carbonation also showed the same trend; when the content of polycarboxylate was 1.2%, the sulphoaluminate carbonation resistance of the cement mortar specimen is the best. At the same time, it can also be found that the carbonation resistance of ordinary Portland cement mortar specimens is better, and the carbonation loss rate is only 2.43%.

#### 3.2.2. Carbonation Depth/Area of Sulphoaluminate Cement Mortar

**Figure 7:**
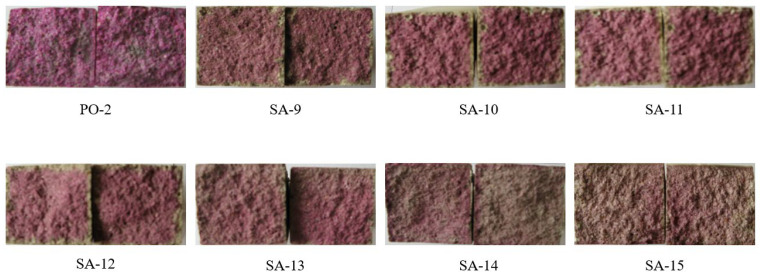
PO-2, SA-9–SA-15 after carbonation with 1% concentration of phenolphthalein indicator.

The cement mortar carbonized sample was split into two equal parts; see Section 3.1.2 for the specific operation method, as shown in Figure 7. According to the schematic diagram in Figure 3, from the surface of the specimen to the discoloration boundary, measure eight distances, obtain the arithmetic mean value as the carbonation depth h, and then calculate the carbonation area S according to Formula (1). The calculation results of the specific carbonation depth and carbonation area are shown in Table 7. From the carbonation depth/area of SA-10 to SA-12, it can be seen that with the increase in the amount of polymer emulsion incorporated, the carbonation depth/area gradually increase, and the carbonation depth/area ratio of SA-10 and SA-11 is SA. The -9 is smaller, while the SA-12 is significantly larger than the SA-9. That is to say, when the addition of polymer emulsion is 5% and 10%, the carbonation resistance of sulphoaluminate cement can be improved. When the addition amount reaches 20%, its carbonation resistance ability is reduced. It can be seen from the carbonation depth/area of SA-13 to SA-15 that the carbonation depth/area is the smallest when the content of polycarboxylic acid is 1.8%. However, the carbonation depth/area of the samples under these three dosages are smaller than those of the blank sample (SA-9); the polycarboxylate increases the carbonation resistance of sulphoaluminate cement under these three dosages. The best resistance to carbonation is when the dosage is 1.8%.

## 4. Conclusions and Outlook

In this paper, research was conducted on the optimal dosage of sulphoaluminate cement paste and mortar mixed with polymer emulsion and polycarboxylic acid. Research was also conducted on the carbonation level and strength before and after carbonation of sulphoaluminate cement paste and mortar-mixed polymer, and polycarboxylic acidwas obtained. The following conclusions were obtained.

(1) For the cement paste, with the increase in polymer emulsion AMPS content, the 3-day strength of the sulphoaluminate cement with a water–cement ratio of 0.3 gradually decreased. When the addition amount is 5% and 10%, the carbonation resistance of sulphoaluminate cement can be improved. In contrast, the addition amount of 20% can reduce its carbonation resistance. The optimum amount of polymer emulsion for the sulphoaluminate cement paste system is 5%. The addition of polycarboxylic acid improves the carbonation resistance of sulphoaluminate cement, and the carbonation resistance is the best when the addition amount is 1.8%.
(2) For the cement mortar, with the increase in the polymer emulsion content, the compressive strength of the sulphoaluminate cement mortar showed a downward trend, but it was higher than that of the blank sample (SA-9). The compressive strength of sulphoaluminate cement increases with the increase in acid content. After carbonation, the compressive strength of each sample decreased, and the changing trend of compressive strength among the groups was consistent with that before carbonation; with the increase in the amount of polymer emulsion incorporated, the carbonation depth/area gradually increased and the polymer emulsion carbonation resistance of sulphoaluminate cement mortar is the best when the addition amount is 5%.

This paper aimed to find a polymer that can be incorporated into sulphoaluminate cement to improve its anti-carbonation ability and to determine the optimal amount of polymer to achieve the best anti-carbonation effect for sulphoaluminate cement concrete. After repeated tests, it was found that the anti-carbonation result of sulphoaluminate cement paste and mortar is the best when the content of polycarboxylic acid is 1.8%. On this basis, it is necessary to try adding other types of polymers and different types of concrete additives to improve the carbonation resistance of sulphoaluminate cement. Follow-up research is urgently needed.

## Figures and Tables

**Table 1:** Composition and properties of sulphoaluminate cement.

Type	CaO (%)	SiO_2_ (%)	Al_2_O_3_ (%)	SO_3_ (%)	MgO (%)	Fe_2_O_3_ (%)	Specific Surface Area (m^2^/kg)
SAC	43.85	16.15	18.53	12.91	2.86	2.62	350
OPC	64.1	20.9	4.8	3.3	1.8	2.6	430

**Table 2:** List of cement paste ingredients.

Abbreviation	Content			
	Cement/g	AMPS (wt%)	Polycarboxylic Acid (wt%)	W/C
PO-1 (1)	OPC/140 g	0	0	0.3
SA-1 (8/17)	OPC/140 g	0	0	0.3
SA-2 (9/18)	SAC/140 g	0	0	0.35
SA-3 (5/14)	SAC/140 g	5	0	0.3
SA-4 (6/15)	SAC/140 g	10	0	0.3
SA-5 (7/16)	SAC/140 g	20	0	0.3
SA-6 (10)	SAC/140 g	0	0.6	0.3
SA-7 (11)	SAC/140 g	0	1.2	0.3
SA-8 (12)	SAC/140 g	0	1.8	0.3

**Table 3:** List of cement mortar ingredients.

Abbreviation	Content				
	Cement/g	AMPS (wt%)	Polycarboxylic Acid (wt%)	W/C	L/S
PO-2 (2)	OPC/450 g	0	0	0.5	0.4
SA-9 (4)	SAC/450 g	0	0	0.5	0.4
SA-10 (19)	SAC/450 g	5	0	0.5	0.4
SA-11 (20)	SAC/450 g	10	0	0.5	0.4
SA-12 (21)	SAC/450 g	20	0	0.5	0.4
SA-13 (22)	SAC/450 g	0	0.6	0.5	0.4
SA-14 (23)	SAC/450 g	0	1.2	0.5	0.4
SA-15 (24)	SAC/450 g	0	1.8	0.5	0.4

**Table 4:** Loss rate of compressive strength of cement paste after carbonation.

Number	PO-1	SA-1	SA-2	SA-3	SA-4	SA-5	SA-6	SA-7	SA-8
The loss rate of compressive strength (%)	1.01	3.06	7.34	5.24	6.58	9.82	5.93	3.98	7.44

**Table 5:** Carbonation depth and carbonation area of PO-1, SA-1–SA-8.

Number	PO-1	SA-1	SA-2	SA-3	SA-4	SA-5	SA-6	SA-7	SA-8
h (mm)	0	0.66	0.76	0.20	0.60	0.80	0.10	0.08	0.05
S (mm^2^)	0	51.06	58.49	15.84	46.56	61.44	7.96	6.37	3.99
Carbonation (%)	0	12.765	16.623	3.96	11.64	15.36	1.99	1.593	0.998

**Table 6:** Loss rate of compressive strength of cement mortar after carbonation.

Number	PO-2	SA-9	SA-10	SA-11	SA-12	SA-13	SA-14	SA-15
The loss rate of compressive strength (%)	2.43	11.96	15.90	18.35	20.41	14.43	15.51	21.58

**Table 7:** Carbonation depth and carbonation area of PO-2, SA-9–SA-15.

Number	PO-2	SA-9	SA-10	SA-11	SA-12	SA-13	SA-14	SA-15
h (mm)	0	2.9	2.2	2.4	3.10	1.6	0.80	0.42
S (mm^2^)	0	198.36	156.64	168.96	209.56	117.76	61.44	32.89
Carbonation (%)	0	49.59	39.16	42.24	52.39	29.44	15.36	8.223
